# A vector-free gene interference system using delaminated Mg–Al-lactate layered double hydroxide nanosheets as molecular carriers to intact plant cells

**DOI:** 10.1186/s13007-023-01021-1

**Published:** 2023-05-08

**Authors:** He Zhang, Xinyu Li, Dong Yu, Junqi Guan, Hao Ding, Hongyang Wu, Qiang Wang, Yinglang Wan

**Affiliations:** 1 Hainan Key Laboratory for Sustainable Utilization of Tropical Bioresources, College of Tropical Crops, Hainan University, Haikou, 570228 China; 2 Key Laboratory of Integrated Pest Management On Tropical Crops, Ministry of Agriculture and Rural Affairs, Environment and Plant Protection Institute, Chinese Academy of Tropical Agricultural Sciences, Haikou, 571101 China; 3 Institute of Vegetables and Flowers, Chinese Academy of Agricultural Sciences, Beijing, 100081 China; 4 College of Environmental Science and Engineering, Beijing Forestry University, 35 Qinghua East Road, Haidian District, Beijing, 100083 China

**Keywords:** RNA delivery, dsRNA, RNAi, Bioconjugates, Target gene expression

## Abstract

**Background:**

The Mg–Al-lactate layered double hydroxide nanosheet (LDH-NS) has shown great potential as an optimal nanocarrier for extensive use in plants. However, previous studies in plant sciences have not provided a clear description of the application for the LDH-NSs-based double-stranded RNA (dsRNA) delivery (LDH-dsRNA) system in different tissues of both model and non-model species.

**Results:**

LDH-NSs were synthesized by using the co-precipitation method, while the dsRNAs targeting genes of interest were prepared in vitro using T7 RNA polymerase. The LDH-dsRNA bioconjugates with a neutral charge were produced by incubating with the mass ratio of LDH-NSs to dsRNA at 3:1, which were then introduced into intact plant cells using three different approaches, including injection, spray, and soak. The LDH-dsRNA delivery method was optimized by inhibiting the expression of the *Arabidopsis thaliana ACTIN2* gene. As a result, soaking *A. thaliana* seedlings in a medium containing LDH-dsRNA for 30 min led to the silencing of 80% of the target genes. The stability and effectiveness of the LDH-dsRNA system were further confirmed by the high-efficiency knockdown of plant tissue-specific genes, including that encoding phytoene desaturase (*PDS*), WUSCHEL (*WUS*), WUSCHEL-related homeobox 5 (*WOX5*), and ROOT HAIR DEFECTIVE 6 (*RHD6*). In addition, the LDH-dsRNA system was employed in cassava, where it was found that the expression of the gene encoding nucleotide-binding site and leucine-rich repeat (*NBS-LRR*) was significantly reduced. As a result, the resistance of cassava leaves to pathogens was weakened. Noteworthy, the injection of LDH-dsRNA into leaves resulted in a significant downregulation of target genes in both stems and flowers, indicating the successful transport of LDH-dsRNA from leaves to other parts of plants.

**Conclusions:**

LDH-NSs have proven to be a highly effective molecular tool for delivering dsRNA into intact plant cells, enabling accurate control of target gene expression.

**Supplementary Information:**

The online version contains supplementary material available at 10.1186/s13007-023-01021-1.

## Background

Genetically modified crops have ushered in a new era of agriculture, offering a potential solution to many of the pressing challenges facing humanity today, including global climate change, food scarcity in developing nations, and pollution caused by traditional petroleum-based farming. The process of genetic engineering in crops requires the incorporation of functional nucleotides into plant cells, which cannot penetrate the plasma membrane spontaneously due to their negative charge [2]. Therefore, the delivery of these molecules through the membrane barrier into cells is a crucial step in genetic engineering within living cells.

In contrast to animal cells, plant cells have peripheral cell walls, which mainly consist of cellulose and pectin polysaccharides. This protective barrier acts to prevent foreign pathogens and particles from entering the cell and damaging the plasma membrane. Various methods of delivering biomolecules to plant cells have been developed to overcome the cell wall barrier. Commonly used methods include organism-based methods such as *Agrobacterium*-mediated delivery [8], chemical methods such as polyethylene glycol-mediated delivery [10, 17], and physical methods such as particle bombardment [7]. However, the limitations associated with these methods, including host specificity, low efficiency, potential tissue damage, and high costs have prompted researchers to explore alternative approaches for delivering biomolecules efficiently [1, 11, 16]. In this regard, nanocarrier-based delivery systems have shown promise for solving these problems by providing a broad-spectrum approach that is non-host specific, low cost, highly efficient, and low in cytotoxicity.

Studies have shown that the intracellular delivery of biomolecules can be achieved through surface spraying, infiltration, or encapsulation using nanoparticles such as gold, starch, mesoporous silica, carbon nanotubes, and LDH-NSs [9, 11, 24, 25]. Nanomaterials have been effectively utilized to deliver various biomolecules into plants, leading to the modification of target gene expression either constitutively or transiently [19]. For instance, single-walled carbon nanotubes (SWNTs) and mesoporous silica nanoparticles (MSNs) have been demonstrated to facilitate the penetration of plasmid DNA into intact plant cells [5, 6]. Magnetic nanoparticles (MNPs) were used as nanocarriers to deliver exogenous genes into the seeds of cotton and maize through their pollen tubes [20, 27], LDH-NSs have been employed to transport various RNA molecules, including dsRNA [13, 22], long noncoding RNA (lncRNA) [15], small interfering RNA (siRNA) [25], and circular RNA (circRNA) [23] into intact cells of diverse plant species. This delivery method has proven effective in regulating the expression of target genes.

Layered double hydroxides (LDHs) belong to a class of pillared clay lamellar materials comprised of positively charged hydroxide layers that are intercalated with anions and water. Lactate intercalated LDHs can be easily delaminated into the water, resulting in LDH-NSs formation. The size of LDH-NSs can be modified within a range of 50–300 nm by using different methods, which affects its efficiency in transporting biomolecules [4, 14, 21]. Studies showed that non-aqueous precipitation methods were used to prepare Mg^2+^–Al^3+^-based LDH-NSs that successfully delivered dsRNA into intact tobacco leaf cells, providing sustained protection against plant viruses [13]. Aqueous methods were used to synthesize Mg^2+^–Al^3+^-based LDH-NSs that can deliver various types of RNAs into plant cells [15, 22, 23, 26]. Furthermore, Wu et al. demonstrated that the Mg^2+^–Al^3+^-based LDH-NSs prepared through aqueous methods exhibit very low cytotoxicity to plants [21]. These findings suggest that LDH-NSs may be an excellent nanocarrier with versatile applications in plant and agricultural sciences.

In this study, LDH-NSs were synthesized using the chemical co-precipitation method with titration temperatures ranging from 0 to 40 °C. The dsRNAs were prepared in vitro using the T7 RNA polymerase and conjugated with the LDH-NSs. The LDH-dsRNA bioconjugates were then used to modify the expressions of target genes in intact plant cells without transgene integration. Overall, we constructed comprehensive guidelines for a vector-free LDH-dsRNA system that can be used across various tissues in both model and non-model species.

## Results

### The vector-free LDH-NS-based gene interference method

**Fig. 1:**
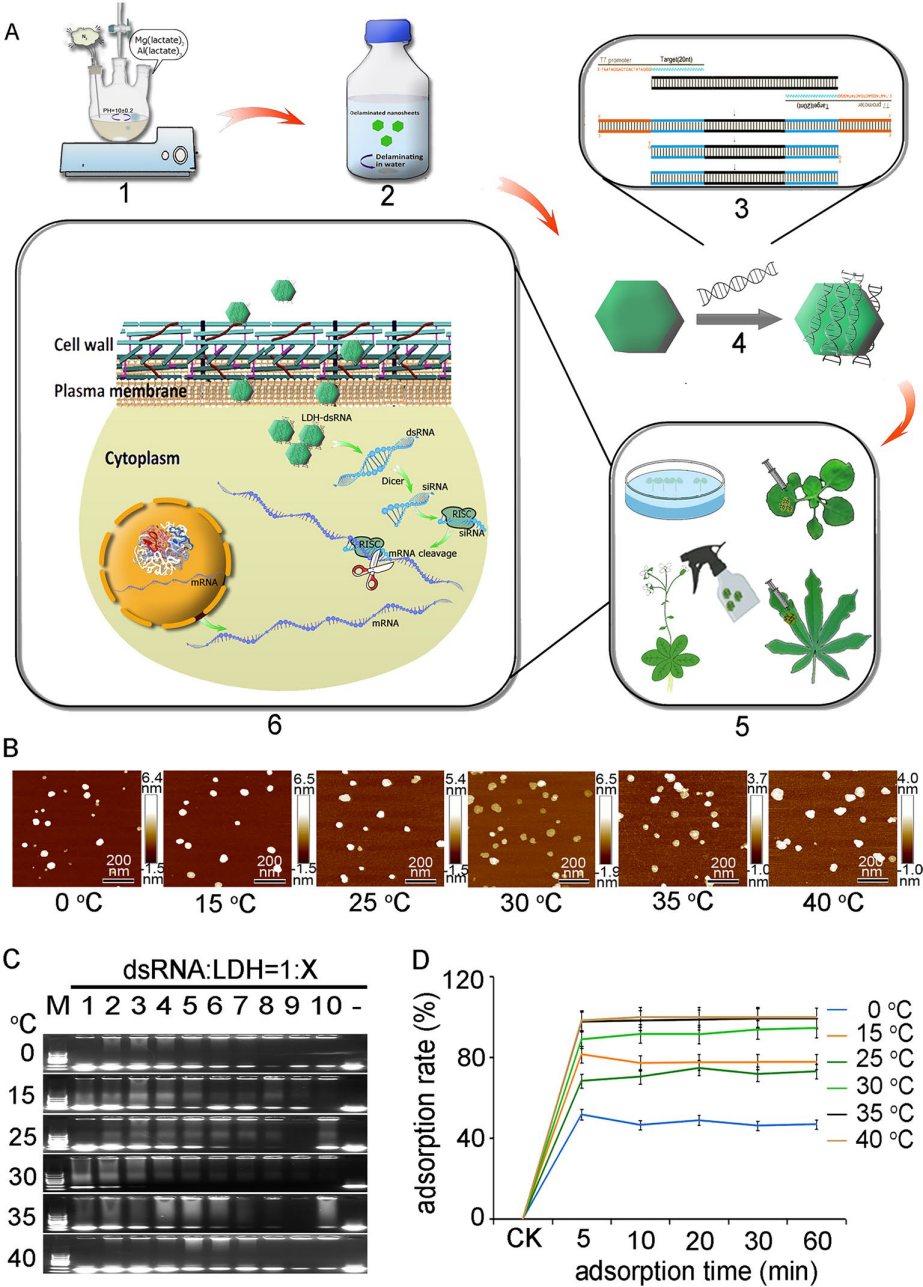
A vector-free method for delivering dsRNA into intact plant cells. **A** Schematic summarizing the steps of the LDH-NS vector-free gene silencing method. 1. LDH-NSs are synthesized from Mg(C_3_H_5_O_3_)_2_·3H_2_O, Al(C_3_H_5_O_3_), and C_3_H_6_O_3_ using co-precipitation. 2. MgAl-LDH is delaminated to form LDH-NSs. 3. Design and in vitro synthesis of dsRNAs corresponding to target genes using T7 RNA polymerase. 4. Adsorption of the dsRNAs onto the LDH-NSs. 5. Delivery of the LDH-dsRNA complex to plant cells. 6. Penetration of the plant by the LDH-dsRNA complex, resulting in the silencing of the target gene. **B** AFM images of delaminated LDH-NSs at different titration temperatures; nanosheets color corresponds to vertical distance. Scale bars = 200 nm. **C** Agarose electrophoresis gels showing the adsorption of dsRNA onto LDH-NSs synthesized at different titration temperatures using various dsRNA:LDH mass ratios. Lanes: M, DL 2000 DNA marker; 1–10, dsRNA:LDH mass ratios of 1:1–1:10; -, control (dsRNA without LDH-NSs). **D** Time curve showing percent dsRNA adsorption onto LDH-NSs synthesized at different temperatures under conditions of dsRNA supersaturation

The method for LDH-dsRNA gene interference presented here involves six sequential steps (Fig. 1A): (1) Preparation of LDH through co-precipitation; (2) Delamination of LDH particles to form LDH-NSs; (3) Synthesis of dsRNA using T7 RNA polymerase in vitro; (4) Bioconjugation of LDH-NSs and dsRNA at a 3:1 mass ratio; (5) Delivery of the LDH-dsRNA bioconjugates into plant cells via different approaches such as soaking, injection or spraying; (6) Penetration of the plant cell by the LDH-dsRNA bioconjugates, leading to silencing of the target gene within the cell. Please refer to the Methods section for more comprehensive details regarding these steps.

### Optimization of LDH-NSs synthesis

The synthesis of LDH-NSs is temperature-dependent, thus changes in temperature can affect its production. To determine the ideal parameters for LDH-NSs synthesis and dsRNA loading, we synthesized LDH-NSs at different titration temperatures. The efficacy of dsRNA loading on LDH-NSs was tested at different dsRNA: LDH-NSs mass ratios and reaction durations. The temperature of the titration reaction was gradually increased from 0 to 40 °C during the co-precipitation of the LDH-NSs. To examine the structures of the resulting LDH-NSs, we used atomic force microscopy (AFM). Image analysis revealed significant differences in vertical distances between the nanosheets and stripping ratios at different titration temperatures (Fig. 1B). The monolayer ratio of LDH-NSs synthesized at 30 °C is 71.94%, which is significantly higher than the ratios obtained at the other five temperatures. The dsRNA_*NbPDS*_ was loaded onto LDH-NSs synthesized at different temperatures by utilizing varying mass ratios of dsRNA to LDH-NSs. The loading efficiency was determined through agarose gel electrophoresis, where light and dark bands indicated low and high loading ratios, respectively. The dsRNA was completely loaded onto LDH-NSs synthesized at 25 °C with a dsRNA: LDH-NSs mass ratio of 1:7, onto LDH-NSs synthesized at 30 °C with a dsRNA: LDH mass ratio of 1:3, and onto LDH-NSs synthesized at 35 °C with a dsRNA: LDH mass ratio of 1:7 (Fig. 1C). However, at synthesis temperatures of 0 °C, 15 °C, and 40 °C, the dsRNA failed to load completely onto LDH-NSs even at a mass ratio of 1:10 (Fig. 1C).

To explore the effects of increased reaction duration on dsRNA adsorption onto the LDH-NSs synthesized at various temperatures, we extended the reaction time incrementally from 0 to 60 min, where 0 min was taken as the control. Despite the synthesis temperature, the negatively charged dsRNA was rapidly adsorbed onto the positively charged LDH-NSs. The total percentage of dsRNA adsorbed onto the LDH-NSs reached a steady state roughly 5 min post-incubation of dsRNA and LDH-NSs, and extending the reaction beyond this point did not increase dsRNA binding to LDH-NSs (Fig. 1D). However, the temperature of LDH-NSs synthesis had a significant influence on dsRNA adsorption, the maximum load of dsRNA on LDH-NSs synthesized at 0 °C 35 °C, and 40 °C being 46.91%, 99.44%, and 99.66%, respectively (Fig. 1D). Overall, our optimization experiments revealed that LDH-NSs synthesized at 30 °C achieved a relatively high maximum dsRNA load (94.59%) at a low dsRNA: LDH mass ratio (1:3). Hence, LDH-NSs synthesized at 30 °C were selected for subsequent experimentation.

### Optimization of LDH-dsRNA delivery method by silencing of *Arabidopsis* AtACTIN2

**Fig. 2:**
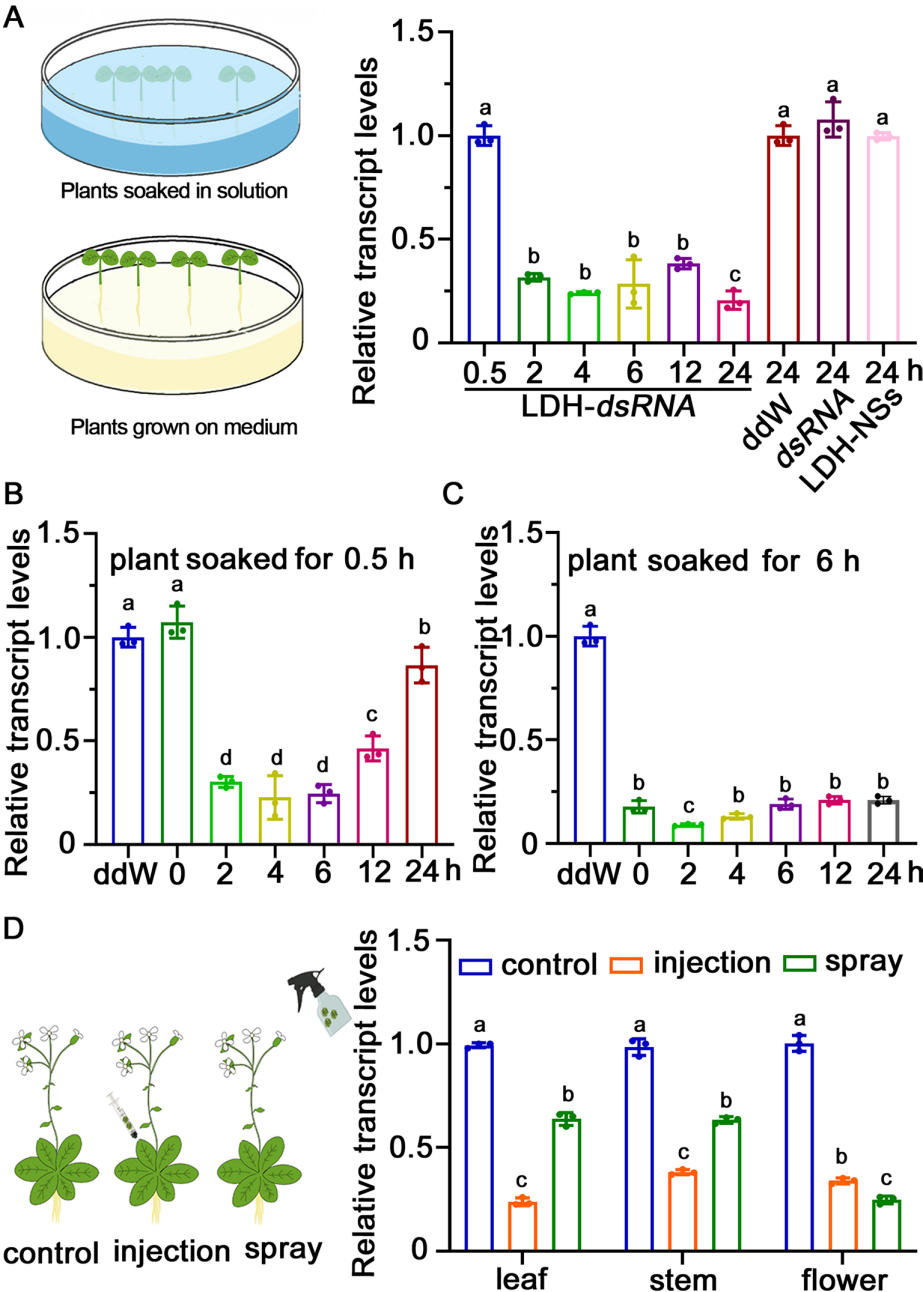
Silencing of *AtACTIN2* in *Arabidopsis* using LDH-NS-mediated dsRNA delivery.** A** The lift picture showed the *Arabidopsis* soaked in a solution containing LDH-dsRNA_*AtACTIN2*_. And put in the solid medium for a time gradient. *AtACTIN2* transcription levels in five-day-old *Arabidopsis* seedlings after 0.5–24 h exposure to dsRNA_*AtACTIN2*_ in liquid medium. Control seedlings were soaked in media containing double distilled water only (ddW), dsRNA_*AtACTIN2*_ only (dsRNA), or LDH-NS only (LDH) for 24 h. **B**,** C** *AtACTIN2* transcription levels in five-day-old *Arabidopsis* seedlings soaked in dsRNA_*AtACTIN2*_-supplemented liquid medium for **B** 0.5 h or **C** 6 h and then cultured on dsRNA-free solid media for 0–24 h. **D** The lift picture showed the *Arabidopsis* that had been injected with LDH-dsRNA_*AtACTIN2*_ or had all their tissues sprayed with it. LDH-dsRNA_*AtACTIN2*_. The tissue-specific transcription level of *AtACTIN2* of mature *Arabidopsis* (leaf, stem, and flower) spray, injection for 24 h. The letters on the bars represent *p* < 0.05, which was calculated using Duncan's multiple range test to indicate statistically significant differences among the various groups

To determine the most effective LDH-dsRNA delivery methods, we performed interference experiments on the *Arabidopsis* housekeeping gene *AtACTIN2*. The dsRNA targeting *AtACTIN2* (dsRNA_*AtACTIN2*_) was produced in vitro by using the T7 RNA polymerase system. The LDH-dsRNA_*AtACTIN2*_ bioconjugates were obtained by incubating with the mass ratio of LDH-NSs to dsRNA_*AtACTIN2*_ at 3:1. Five-day-old *Arabidopsis* seedlings were soaked in a solution supplemented with LDH-NSs-dsRNA_*AtACTIN2*_ for 24 h. Results showed that *AtACTIN2* expression levels did not show a significant difference in comparison with the control group after soaking for 0.5 h. The expression levels of *AtACTIN2* significantly decreased after soaking for 2–24 h compared to the control group, with a further significant decrease observed between 12 and 24 h. At the end of 24 h, the expression levels of *AtACTIN2* were less than 20% of that in the control group (Fig. 2A).

The seedlings were soaked in the liquid culture medium supplemented with LDH-dsRNA_*AtACTIN2*_ for 0.5 and 6 h, followed by transfer to a dsRNA-free solid culture medium over time (Fig. 2B, C). When seedlings were soaked in the dsRNA-supplemented medium for 0.5 h before being cultured on dsRNA-free solid media, *AtACTIN2* expression was significantly downregulated compared to the control at 2–12 h. Notably, the expression level of *AtACTIN2* at 24 h was significantly higher than that at 2–12 h, while it was significantly lower than that at 0 h (Fig. 2B). At 6 h of dsRNA exposure, there was a significant downregulation of *AtACTIN2* compared to the control. This downregulation persisted until the end of the experiment at 24 h. (Fig. 2C).

To verify the efficacy of gene silencing using different methods, we administered LDH-dsRNA_*AtACTIN2*_ to the flowering *A. thaliana* Col-0 through both spray and injection methods. Regardless of the methodology employed, a significant decrease in *AtACTIN2* transcription abundance was observed in the leaves, stems, and flowers when compared to the control (Fig. 2D). These results suggested that dsRNA delivery methods including soak, spray, and injection are viable options for use under different experimental conditions.

### Silencing of *Arabidopsis* AtPDS3, AtWUS, AtWOX5, and AtRHD6

**Fig. 3:**
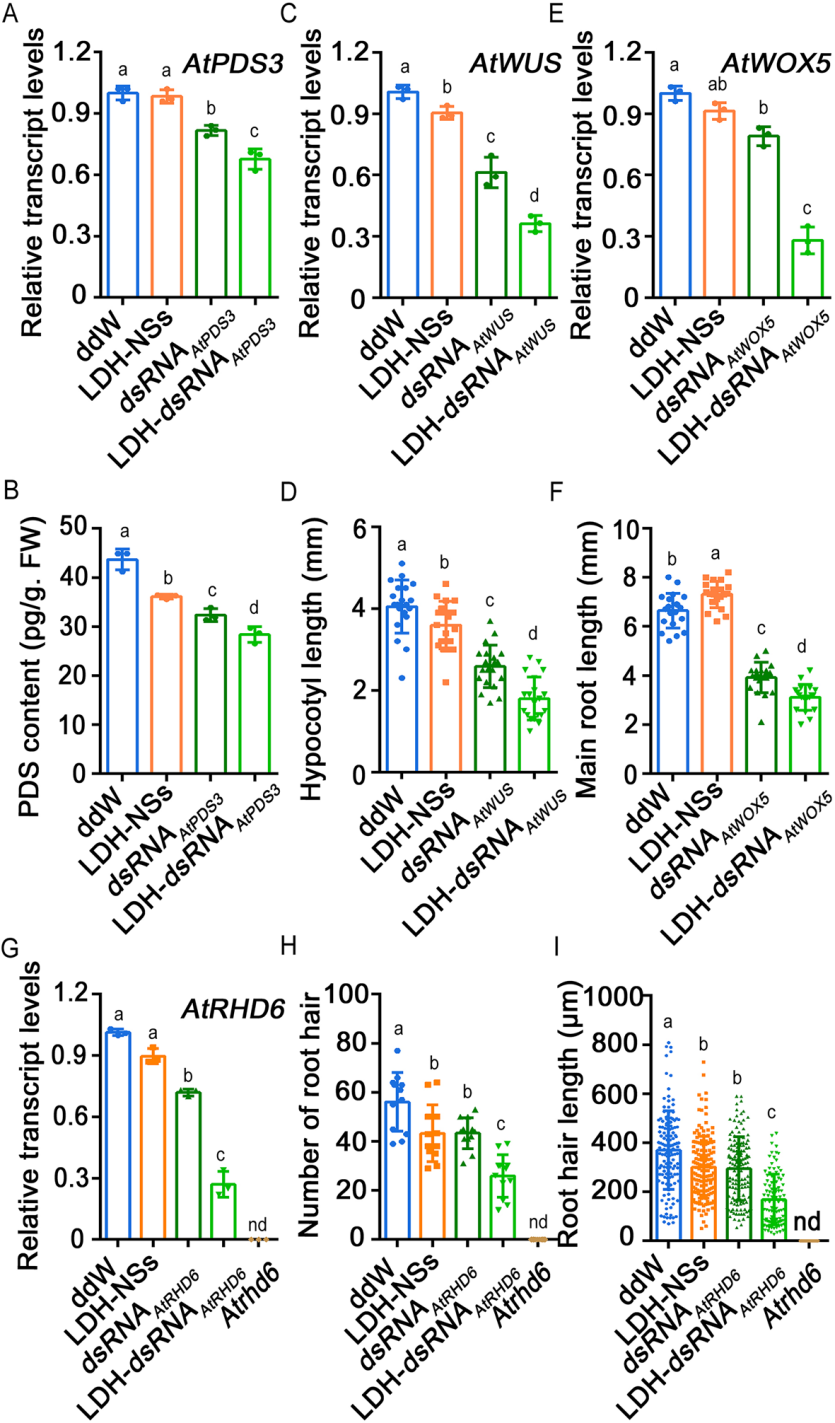
Silencing of *AtPDS3*, *AtWUS*, *AtWOX5*, and *AtRHD6* in *Arabidopsis* using LDH-mediated dsRNA delivery. **A**,** B**: **A** Relative *AtPDS3* transcription levels and **B** PDS protein contents in *AtPDS3*-silenced and control *Arabidopsis* leaves. **C**, **D**: **C** Relative *AtWUS* transcription levels in the SAM and **D** hypocotyl length of *AtWUS*-silenced and control *Arabidopsis* seedlings. **E**, **F**: **E** Relative *AtWOX5* transcription levels in the root tip and **F** main root of *AtWOX5*-silenced and ddW control *Arabidopsis* seedlings*.* **G**–**I**: **G** Relative *AtRHD6* transcription in the root, **H** the number of root hairs, and **I** the length of the root hairs of *AtRHD6*-silenced and ddW control *Arabidopsis* seedlings. Line *Atrhd6* is an *Arabidopsis* mutant without root hairs. The letters on the bars represent *p* < 0.05, which was calculated using Duncan's multiple range test to indicate statistically significant differences among the various groups. nd, not detected

To further validate the effectiveness of the LDH-NS-based dsRNA delivery method in various parts of model plant *Arabidopsis*, we employed LDH-dsRNA bioconjugates to silence the *AtPDS3* gene in the leaves, *AtWUS* gene in the shoot apical meristems (SAMs), *AtWOX5* gene in the root tips, and *AtRHD6* gene in the roots, respectively. The expression level of *AtPDS3* was significantly downregulated after treatment with both dsRNA_*AtPDS3*_ and LDH-dsRNA_*AtPDS3*_ compared to double distilled water (ddW) and LDH-NS treatment. Moreover, *AtPDS3* gene expression level was lower in LDH-dsRNA_*AtPDS3*_ treated plants than those treated with dsRNA_*AtPDS3*_ (Fig. 3A). These results were consistent with the quantitative analysis of PDS protein content (Fig. 3B). As shown in Fig. 3C, *AtWUS* transcription level in the SAM of 5-day-old seedlings treated with dsRNA_*AtWUS*_ and LDH-dsRNA_*AtWUS*_ exhibited a significant decrease compared to the ddW treatment group. Additionally, *AtWUS* expression was significantly downregulated after LDH-dsRNA_*AtWUS*_ treatment in comparison to dsRNA_*AtWUS*_ treatment (Fig. 3C). Differences in hypocotyl length were consistent with *AtWUS* gene expression patterns (Fig. 3D). As shown in Fig. 3E, the transcription levels of *AtWOX5* were significantly reduced in both dsRNA_*AtWOX5*_ and LDH-dsRNA_*AtWOX5*_ treatment groups compared to the ddW treatment group. *AtWOX5* transcription level in the LDH-dsRNA_*AtWOX5*_ group was significantly lower than that in the dsRNA_*AtWOX5*_ group. Compared with the ddW treatment group, main root growth was significantly inhibited in both dsRNA_*AtWOX5*_ and LDH-dsRNA_*AtWOX5*_ treatment groups. The main root in the LDH-NSs group was significantly longer than that in the ddW group, while it was significantly shorter in the LDH-dsRNA_*AtWOX5*_ group compared to the dsRNA_*AtWOX5*_ group (Fig. 3F). Additionally, the GFP fluorescence intensity was significantly lower in the root tips of the AtWOX5: GFP transgenic line treated with LDH-dsRNA_*AtWOX5*_ compared to those treated with ddW, LDH-NS, and *dsRNA*_*AtWOX5*_ (Additional file 1: Fig. S2A and B). As shown in Fig. 3G, *AtRHD6* transcription levels were significantly decreased in 3-day-old seedlings treated with dsRNA_*AtRHD6*_ and LDH-dsRNA_*AtRHD6*_ compared to the ddW treatment group. The seedlings treated with LDH-dsRNA_*AtRHD6*_ had significantly fewer and shorter root hairs than those in all other treatment groups (Fig. 3H and I), indicating successful inhibition of root hair initiation and elongation. Overall, these results verified that LDH-NSs are capable of effectively delivering dsRNA to different parts of Arabidopsis, leading to a characteristic phenotypic alteration.

### Silencing of tobacco NbPDS, NbWUS, and NbWOX5

**Fig. 4:**
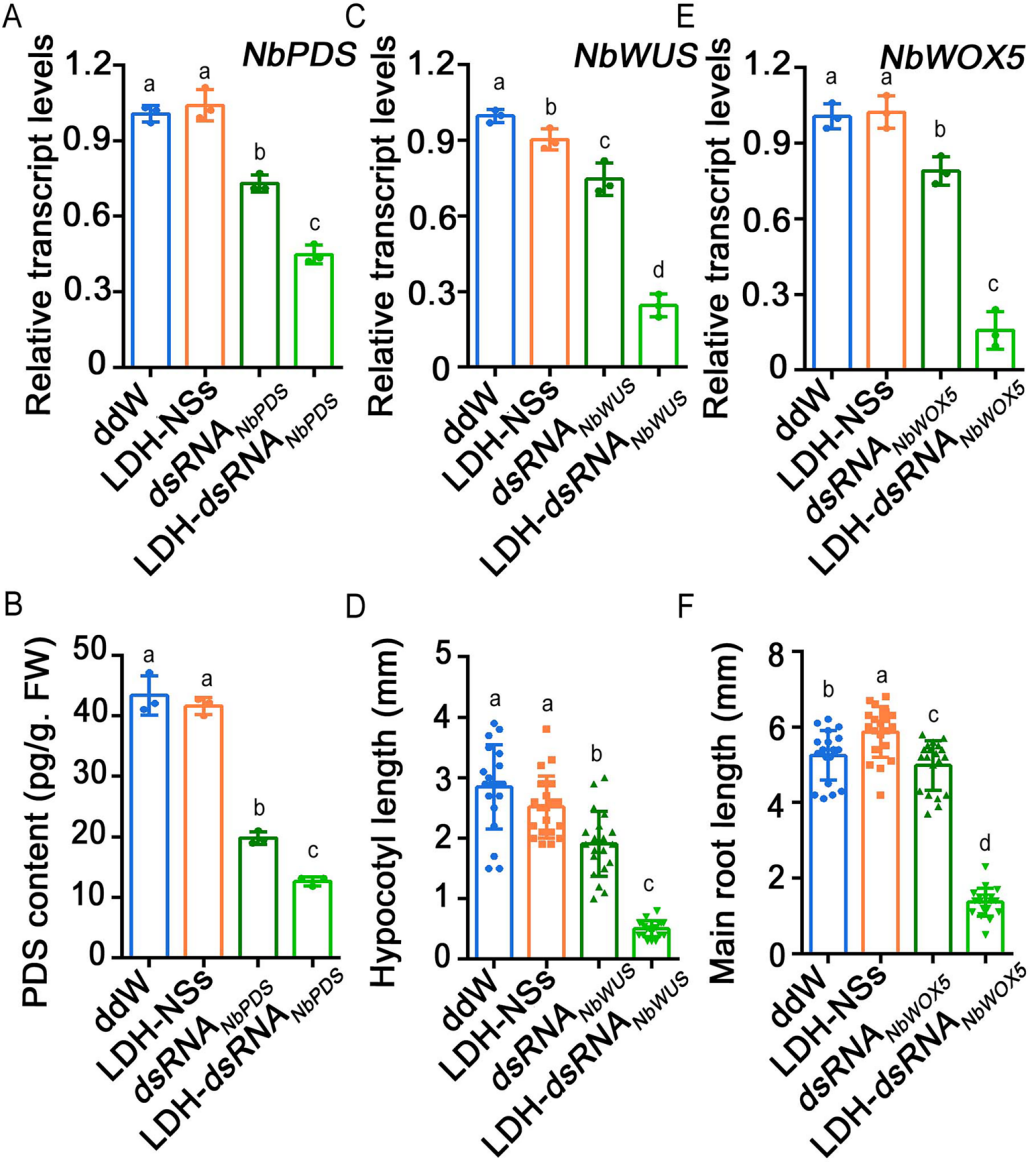
Silencing of *NbPDS*, *NbWUS*, and *NbWOX5* in tobacco using LDH-mediated dsRNA delivery. **A**, **B**: **A** Relative *NbPDS* transcription levels and **B** PDS protein contents in the *NbPDS*-silenced and ddW control tobacco leaves. **C**,** D**: **C** Relative *NbWUS* transcription levels in the SAM and **D** hypocotyl length of *NbWUS*-silenced and ddW control tobacco seedlings. **E**, **F**: **E** Relative *NbWOX5* transcription levels in the root tip and **F** main root of *NbWOX5*-silenced and ddW control tobacco seedlings*.* The letters on the bars represent *p* < 0.05, which was calculated using Duncan’s multiple range test to indicate statistically significant differences among the various groups

To investigate the potential of LDH-NSs to incorporate dsRNAs targeting genes in other plant species, we employed LDH-dsRNA bioconjugates to silence *NbPDS*, *NbWUS*, and *NbWOX5* genes in tobacco. As shown in Fig. 4A, *NbPDS* transcription levels were significantly decreased in both the dsRNA_*NbPDS*_ and LDH-dsRNA_*NbPDS*_ treatment groups compared to all other treatment groups. Additionally, the expression levels of *NbPDS* were significantly lower in the LDH-dsRNA_*NbPDS*_ treatment group than that in the dsRNA_*NbPDS*_ treatment group. These results were consistent with the quantitative analysis of PDS protein content (Fig. 4B). As shown in Fig. 4C and D, the expression levels of *NbWUS* in the SAM region of tobacco were notably reduced and resulted in a significant decrease in hypocotyl length within the dsRNA_*NbWUS*_ and LDH-dsRNA_*NbWUS*_ treatment groups compared to all other treatment groups. In comparison to the dsRNA_*NbWUS*_ treatment group, the LDH-dsRNA_*NbWUS*_ treatment group exhibited a significant reduction in both *NbWUS* transcription levels and hypocotyl length (Fig. 4C and D). As shown in Fig. 4E, the transcription levels of *NbWOX5* in tobacco root tips showed a significant decrease in both dsRNA_*NbWOX5*_ and LDH-dsRNA_*NbWOX5*_ groups compared to ddW and LDH-NSs groups*.* Moreover, *NbWOX5* exhibited the lowest expression levels in the LDH-dsRNA_*NbWOX5*_ treatment groups among all four treatment groups. Notably, the LDH-dsRNA_*NbWUS*_ group showed significant inhibition in the growth of the main root compared to the dsRNA_*NbWUS*_ group. (Fig. 4F). Overall, these findings suggest that LDH-NSs were able to effectively deliver dsRNA into different parts of tobacco.

### Silencing of cassava MeLRRs

**Fig. 5:**
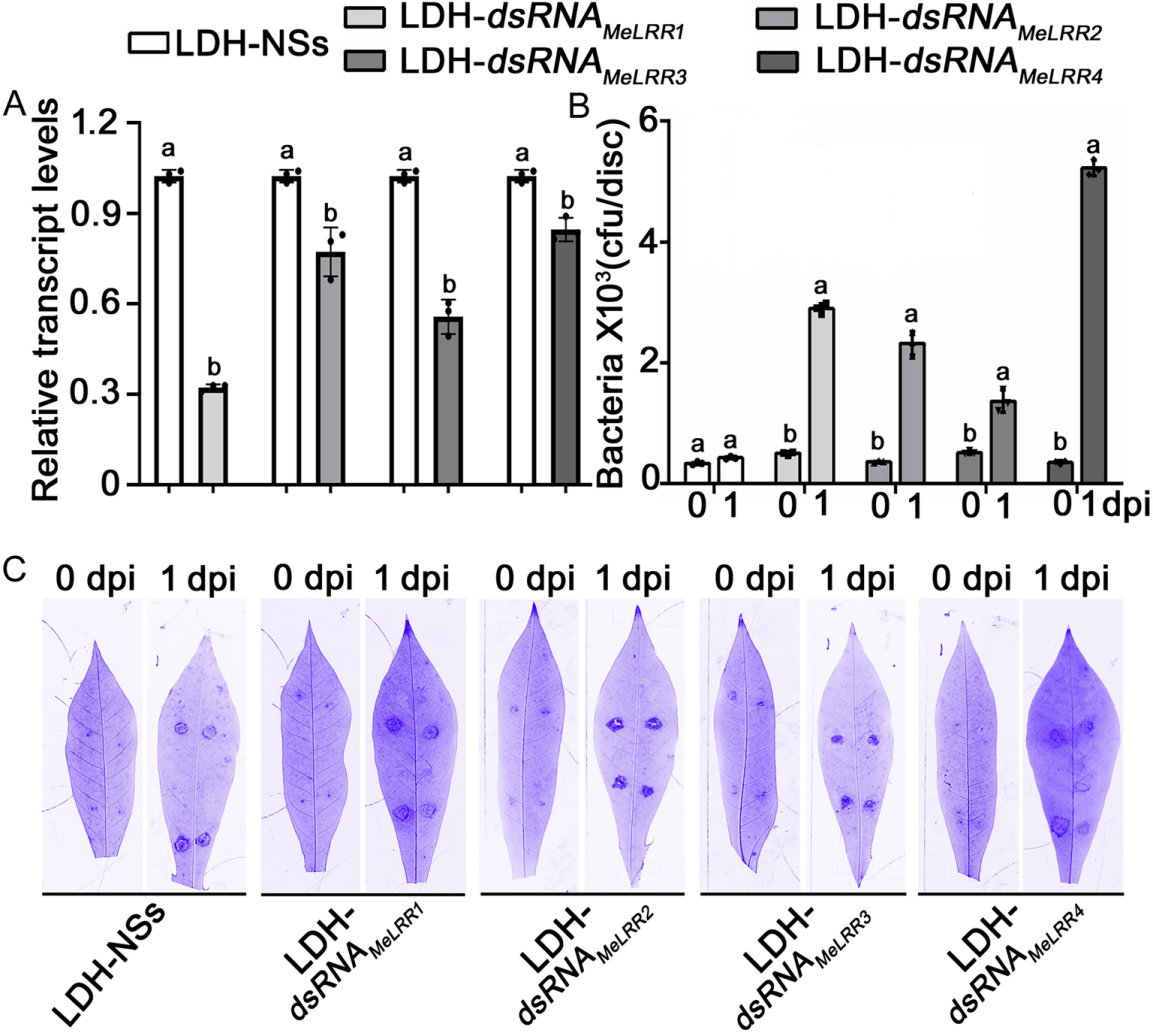
LDH-NS-mediated silencing of *MeLRRs* reduced the resistance of cassava to cassava bacterial blight. **A** The relative transcription levels of *MeLRRs* in *MeLRR-*silenced and LDH control cassava leaves. **B** The number of *Xanthomonas axonopodis* pv. *Manihotis* (*Xam*) populations in the *MeLRR-*silenced and the LDH control cassava leaves; *Xam* is the pathogenic bacterium causing cassava bacterial blight. NS, not significantly different. **C** Cassava leaves stained with Coomassie brilliant blue were imaged using a Fusion FX7-826 apparatus (Vilber Lourmat, France). The letters on the bars represent *p* < 0.05, which was calculated using a Student’s *t*-test to indicate statistically significant differences among the various groups

Previous research has confirmed that the gene encoding cassava leucine-rich repeat (*MeLRR*) is responsible for conferring cassava resistance to cassava bacterial blight (*Xanthomonas axonopodis* pv. *manihotis*, *Xam*) [26]. To investigate the effectiveness of the LDH-dsRNA system in combating cassava bacterial blight, cassava leaves were infiltrated with both LDH-dsRNA_*MeLRRs*_ and LDH-NS. As shown in Fig. 5A, the transcription levels of the *MeLRR* gene in cassava leaves showed a significant decrease following treatment with LDH-dsRNA_*MeLRRs*_ compared to treatment with LDH-NS. Of these, the expression change of *MeLRR1* gene is the largest, while that of *MeLRR4* gene is the smallest. The expression change of *MeLRR3* gene is smaller than that of *MeLRR1* gene, but larger than that of *MeLRR2* gene. As shown in Fig. 5B, the cassava leaves that were infiltrated with LDH-dsRNA_*MeLRRs*_ had significantly higher bacterial abundance than those that were infiltrated with LDH-NS. The larger disease spots indicate weaker disease resistance. Thus, the silencing of the *MeLRRs* reduces the resistance of cassava leaves to *Xam* (Fig. 5C).

## Discussion

Plant cell walls hinder the efficiency and broad-spectrum applicability of conventional gene delivery methods. New biomolecular delivery technologies for plants are therefore urgently needed. In previous studies, we demonstrated that MgAl-LDH-NSs could be used to deliver single-stranded DNA (ssDNA) and genomic DNA into plants [3, 4, 21]. Mg–Al-LDH-NSs are cationic nanoparticles that deliver negatively charged dsRNA into the cell via electrostatic interactions, subsequently resulting in gene silencing. We also previously showed that the temperature of the titration reaction during LDH-NSs co-precipitation determined the capacity of the LDH-NSs to adsorb DNA, as well as its zeta potential and particle size [21].

In this study, we first aimed to identify the optimal titration temperature for LDH-NSs co-precipitation. We found that Mg–Al-LDH-NSs synthesized at a titration temperature of 30 °C were optimal for dsRNA adsorption. In addition, AFM images showed that LDH-NSs synthesized at 30 °C had the highest specific surface area which allows more biomacromolecules can be adsorbed and delivered.

Using a housekeeping gene (*ACTIN2*) in the model plant *Arabidopsis,* we aimed to optimize LDH-dsRNA treatment duration and conditions. We found that *AtACTIN2* was downregulated after 2 h of exposure to the LDH-dsRNA complex and that exposure to the LDH-dsRNA complex for as little as 0.5 h was sufficient to interfere with target gene expression silencing efficiency of about 80%, with effects first noticeable 2 h after exposure. Importantly, target gene expression in the seedlings soaked in the LDH-dsRNA solution for 0.5 h increased after 24 h of growth on an unmodified medium. This suggested that the LDH-dsRNA complex might potentially be useful for transient gene silencing in plant cells, without altering the genetic material of the cell. In mature *Arabidopsis* plants, target gene expression was reduced significantly compared to controls whether LDH-dsRNA was applied via spray or injection. However, the injection was significantly more effective than spraying.

Because housekeeping gene expression is uniform, stable, and relatively high across all plant tissues, we also tested the efficacy of LDH-NS-mediated dsRNA delivery against several tissue-specific genes in the model plants *Arabidopsis* and tobacco: *PDS, WUS*, *RHD6*, and *WOX5*, which are particularly strongly expressed in the leaves, SAMs, roots, and root apical meristems, respectively. However, because we did not restrict our gene expression analyses to the tissues specifically expressing each gene, the apparent efficiency of this method seemed lower for the tissue-specific genes than for the housekeeping gene. The study found that treatment with LDH-NSs led to significant suppression of *AtWOX5* expression, suggesting that LDH synthesized with metallic elements may have an impact on the development of root tips.

To test whether vector-free LDH-NSs-mediated dsRNA delivery might be useful in agricultural applications, we attempted to silence genes in cassava, an economically important plant. Injection of LDH-dsRNAs targeting *MeLRRs* significantly reduced the resistance of the cassava-to-cassava bacterial blight (*Xam*), suggesting that LDH-NS-mediated gene silencing might have wide application prospects in the agricultural industry. The vector-free LDH-NSs-mediated dsRNA delivery method successfully significantly repressed the expression of all of these genes. Our results showed that the LDH-NSs rapidly adsorbed dsRNA and that the dsRNA was efficiently internalized by the mature walled cells of both model plants (*Arabidopsis* and tobacco) and non-model plants (the economically important cassava).

Sustainable agricultural production practices are facing urgent problems including how to mitigate the reliance on the use of agrochemicals, meanwhile enhancing the quality and yields of agri-foods. The emergence of nanotechnology has shown promise for solving these problems. LDH-NSs-based transient gene silencing system has the advantages of being biodegradable, non-host specificity, non-genome integration, and more simplified operation than conventional transgenic techniques. These advantages demonstrate the great potential of this system for application in agricultural practices, such as pest and disease control by regulating gene expression, while at the same time promoting the quality and yields of agri-foods by loading bioactive ingredients.

## Conclusion

In conclusion, compared to traditional gene-silencing techniques targeting plant cells, such as *Agrobacterium*-method transformation and particle bombardment, gene silencing using LDH-NSs as dsRNA nanocarriers has several unique advantages: (i) LDH-NSs mediated gene silencing is vector free with high interfering efficiency up to 80%. The silencing of the target gene(s) can be transient, without causing permanent genetic modification and long-term effects. (ii) LDH-NSs mediated gene silencing is broad spectrum. This method was successful in several kinds of (Arabidopsis, tobacco, and cassava) plant cells tested herein, irrespective of plant age (i.e. seedling or mature), tissue, or species. However, the success of this technique in woody plants and plants with waxy cuticles remains to be confirmed. (iii) LDH-NSs-mediated gene silencing is simple, inexpensive, and time-saving. LDH-NSs can be produced in any laboratory without special equipment, while dsRNAs are readily synthesized using the widely available T7 RNA-polymerase system. Therefore, we believe that LDH-NSs-mediated dsRNA delivery will be of great use to researchers in the plant and agricultural sciences.

## Methods

### Preparation of the LDH-NSs

Delaminated LDH-NSs were chemically synthesized from Mg(C_3_H_5_O_3_)_2_·3H_2_O, Al(C_3_H_5_O_3_), and C_3_H_6_O_3_ as previously described method [18]. To optimize the preparation of LDH-NSs, the slurry of wet LDH-NSs (with a pH of 10) was stirred while maintaining a constant N_2_ atmosphere. The LDH-NSs titration procedure was conducted at various temperatures using the co-precipitation method, including 0 °C, 15 °C, 25 °C, 30 °C, 35 °C, and 40 °C. To avoid the adsorption of CO_2_ by reaction products, N_2_ shall be continuously injected. The slurry was then centrifuged (5000 rpm 10 min), and the precipitate was rinsed with decarbonated water three times. The dried precipitate was transferred to decarbonated water in a concentration of 1 g L^−1^ and stirred magnetically at 350 rpm.

### Analysis of zeta potential

The zeta potential analysis of delaminated LDH-NSs was conducted using a Malvern 2000 zeta potential analyzer (Malvern Instruments, Malvern, UK) following the previously described methods [3, 18].

### AFM imaging

The Bruker Dimension AFM system (version 1.80R1, Bruker Company, Billerica, USA) was used for the analysis of AFM images following the previously described methods [3, 18].

### Synthesis of dsRNAs in vitro

The sequences of the target genes of *A. thaliana* (including *AtACTIN2*, *AtPDS3*, *AtWUS*, *AtWOX5*, and *AtRHD6*), *N. benthamiana* (including *NbPDS*, *NbWUS*, and *NbWOX5*)*,* and *Manihot esculenta* (including *MeLRR1*, *MeLRR2*, *MeLRR3*, and *MeLRR4*) were download from NCBI GenBank website (http://www.ncbi.nlm.nih.gov/) (Additional file 1: Table S1). The gene-specific primers were designed by Primer Premier 6.0 (PREMIER Biosoft International, San Francisco, USA Additional file 1: Table S2). The dsRNAs with lengths of 117–368 bp were designed using SiDirect (http://sidirect2.rnai.jp/). The dsRNAs were synthesized in vitro using the HiScribe T7 Quick High Yield RNA Synthesis Kit (NEB, USA) (Additional file 1: Fig. S1). The primers are listed in Additional file 1: Table S2.

### Preparation of LDH-dsRNA bioconjugates

The adsorption experiments were performed in batches by mixing 1 mL LDH-NSs stock solution (100 μg mL^−1^) with varying amounts of dsRNA stock solution (100 μg mL^−1^), resulting in the final mass ratio of 1:1, 1:2, 1:3, 1:4, 1:5, 1:6, 1:7, 1:8, 1:9, and 1:10. LDH-NSs were conjugated with each dsRNA in RNase free water at room temperature for 5 min.

### Optimization of LDH-mediated dsRNA_AtACTIN2_ delivery system

The LDH-dsRNA targeting *AtACTIN2* was co-cultured with 5-day-old *Arabidopsis* in tissue culture plates for 0.5 and 6 h of soaking. After being soaked in a solution containing LDH-*dsRNA*_*AtACTIN2*_, *Arabidopsis* seedlings were transferred to a solid tissue culture plate and kept for 0, 2, 4, 6, 12, and 24 h. The dsRNAs (final concentration 15 mg L^−1^) were incubated with the LDH-NSs (final concentration 45 mg L^−1^) at room temperature for 5 min prior to the experiment. The transcription levels of *AtACTIN2* were measured using qRT-PCR after 0.5, 2, 4, 6, 12, and 24 h of soaking.

### Comparison of different methods for introducing LDH-dsRNA_AtACTIN2_ to *Arabidopsis*

To evaluate the effectiveness of LDH-dsRNA_*AtACTIN2*_ in the different parts of *Arabidopsis*, we applied it to *Arabidopsis* plants using both spray and injection methods. After 12 h, we measured the transcription levels of *AtACTIN2* in various tissues using qRT-PCR.

### Analyzing the effectiveness of the LDH-dsRNA in *A. thaliana*

Arabidopsis seeds (Col-0 and *AtWOX5: GFP*) were surface sterilized and cultured in 6-well tissue culture plates containing ddW, LDH-NS, dsRNA_*AtRHD6/ AtWOX5/ AtWUX*_ or LDH-dsRNA_*AtRHD6/ AtWOX5/AtWUX*_ for three days, respectively. The culture solution was replaced every 24 h. Root hairs were examined using an optical microscope (Nikon, Japan). Hypocotyl length, main root length, and root hair length were measured from images of each sample using ImageJ software (version 1.53q). The fluorescence of GFP was captured by confocal microscopy at Ex = 488 nm and Em = 520 nm (SP8, Leica, Germany). The average fluorescence intensity values of GFP in the root tips were measured using ImageJ (version 1.53q). In addition, the injection method was employed to silence *AtPDS3* in the leaves of 30-day-old *A. thaliana*.

### Analyzing the effectiveness of the LDH-dsRNA in *N. benthamiana*

Tobacco seeds were surface-sterilized and then cultured in 6-well tissue culture plates containing ddW, LDH-NS, dsRNA_*NbPDS/NbWUS/ NbWOX5*,_ and LDH-dsRNA_*NbPDS/NbWUS/ NbWOX5*_ for 3 days, respectively. The culture solution was replaced every 24 h. The expression levels of target genes were measured by using qRT-PCR. The contents of PDS proteins were measured using enzyme-linked immunosorbent assay (ELISA) kits (Jiangsu Meimian Industrial Co., China). In addition, the injection method was employed to silence *NbPDS* in the leaves of 30-day-old *N. benthamiana*.

### Analyzing the effectiveness of the LDH-dsRNA in cassava leaves

The South China 124 (SC124) variety of cassava (*M. esculenta*) was cultivated in a greenhouse under a 16 h light (28 °C) and 8 h dark (22 °C) cycle. LDH-dsRNA_*MeLRR*_ bioconjugates were obtained by incubating 90 mg L^−1^ LDH-NS with 30 mg L^−1^ dsRNA. Leaves of 4-week-old cassava plants were subjected to syringe infiltration with a solution containing LDH-dsRNA_*MeLRR1*_, LDH-*dsRNA*_*MeLRR2*_, LDH -dsRNA_*MeLRR3*_, and LDH-dsRNA_*MeLRR4*_, respectively. To serve as a negative control, leaves were treated with a solution containing 90 mg L^−1^ LDH-NS using syringe infiltration. The infiltrated plants were then transferred to a greenhouse. The expression level of *MeLRR* genes was measured after 3 days post-infiltration. The leaves were then syringe-infiltrated with the pathogenic bacterium *Xam* at a concentration of 4 × 10^6^ CFU (colony-forming units) mL^−1^. The numbers of *Xam* colonies were measured using the colony counting method at 0 and 1 days post-*Xam* infiltration [26]. Cassava leaves infected with *Xam* were treated with Coomassie brilliant blue stain and subsequently visualized using a Fusion FX7-826 apparatus (Vilber Lourmat, France) [26].

### Gene expression analysis

Total RNA was extracted from plant samples using the RNAprep Pure Plant Kit (Tiangen, China). The total RNA was reversely transcribed into cDNA using the FastQuant RT Kit with gDNase (Tiangen, China). Quantitative real-time reverse-transcription PCR (qRT-PCR) was performed on an Applied Biosystems QuantStudio 6 Flex Real-Time PCR System using the UltraSYBR mixture (Beijing CoWin Biotech., China) and gene-specific primers (Additional file 1: Table S3). The relative expression levels were determined using the previously described 2^–ΔΔCt^ method [12].

### Statistical analysis

In bar plots, data are presented as mean ± standard error (SE). Friedman test with two-sided Dunn’s multiple comparisons test was performed to compare multiple groups. A paired two-tailed *t*-test was used to compare the expression level of cassava *MeLRR*. Different letters indicate *p* < 0.05. All statistical analyses were conducted using GraphPad (vison 6.02, USA).

## Supplementary Information

**Additional file 1**: **Figure S1**. Flowchart for in vitro artificial synthesis dsRNA. **Figure S2**. Laser scanning confocal microscope observations of GFP intensity after LDH-NS-mediated silencing of AtWOX5: GFP. A Observations of green fluorescent cells in the root tips of the Arabidopsis AtWOX5: GFP. ddW, double distilled water. Scale bars = 50 µm. B Area of green fluorescence in the root tips of the Arabidopsis AtWOX5: GFP. The letters on the bars represent p < 0.05, which was calculated using Duncan's multiple range test to indicate statistically significant differences among the various groups. **Table S1**. Information of the genes in this study. **Table S2**. Specific primers for in vitro-synthesized dsRNA in this study. **Table S3**. Primers used in qRT-PCRs in this study.

## Data Availability

Not applicable.
