# Supplementary material for: A vector-free gene interference system using delaminated Mg–Al-lactate layered double hydroxide nanosheets as molecular carriers to intact plant cells
Source: Plant Methods. 2023 May 8;19:44. doi: 10.1186/s13007-023-01021-1 (PMC10165820; doi:10.1186/s13007-023-01021-1)
Supplement: Supplementary file 1 — Additional file 1: Figure S1. Flowchart for in vitro artificial synthesis dsRNA. Figure S2. Laser scanning confocal microscope observations of GFP intensity after LDH-NS-mediated silencing of AtWOX5: GFP. A Observations of green fluorescent cells in the root tips of the Arabidopsis AtWOX5: GFP. ddW, double distilled water. Scale bars = 50 µm. B Area of green fluorescence in the root tips of the Arabidopsis AtWOX5: GFP. The letters on the bars represent p < 0.05, which was calculated using Duncan's multiple range test to indicate statistically significant differences among the various groups. Table S1. Information of the genes in this study. Table S2. Specific primers for in vitro-synthesized dsRNA in this study. Table S3. Primers used in qRT-PCRs in this study. [file 13007_2023_1021_MOESM1_ESM.docx]

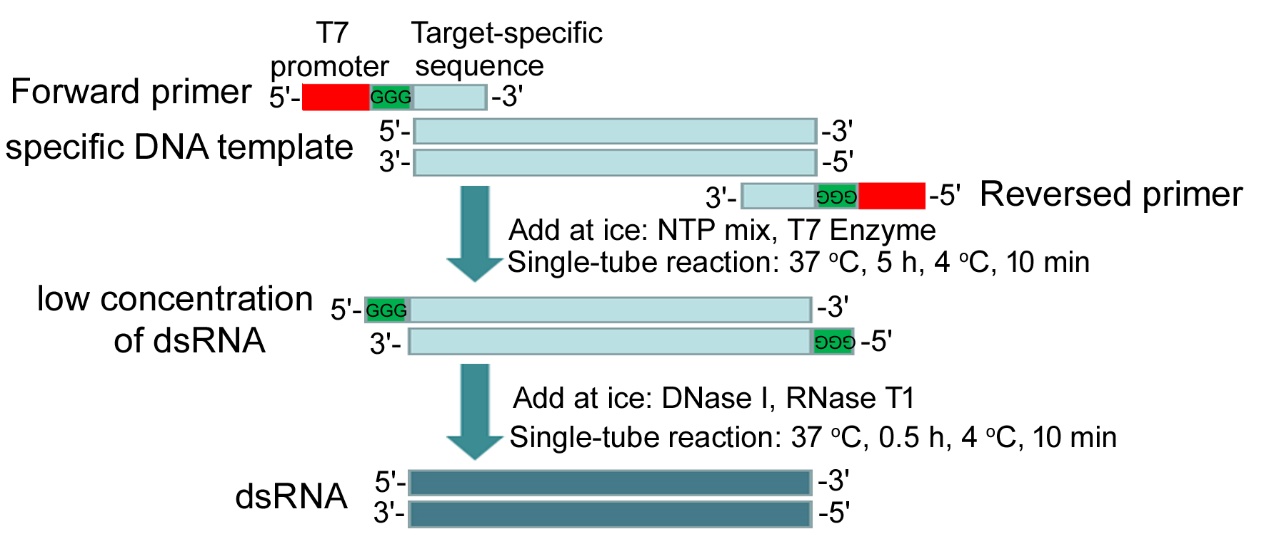


**Supplementary Figure S1** Flowchart for *in vitro* artificial synthesis dsRNA.

**
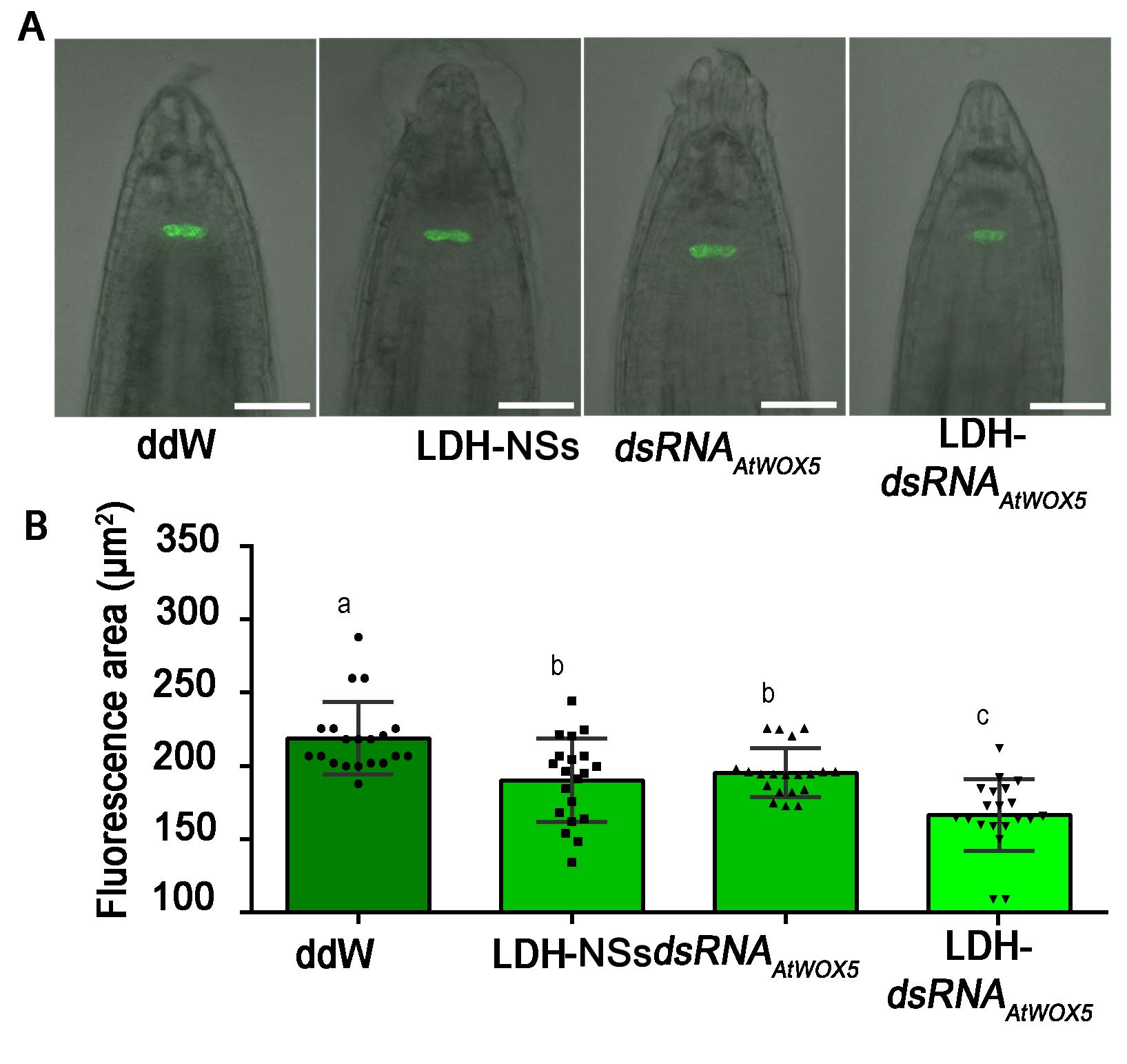
**

**Supplementary Figure S2 Laser scanning confocal microscope observations of GFP intensity after LDH-NS-mediated silencing of *AtWOX5: GFP*. A**: Observations of green fluorescent cells in the root tips of the *Arabidopsis* *AtWOX5: GFP*. ddW, double distilled water. Scale bars = 50 µm. **B**: Area of green fluorescence in the root tips of the *Arabidopsis* *AtWOX5: GFP*. The letters on the bars represent *p* < 0.05, which was calculated using Duncan's multiple range test to indicate statistically significant differences among the various groups.

**Supplementary Table S1** Information of the genes in this study

| **Gene name** | **Product** | **GenBank Number** | **Species** | **CDS length**  **(bp)** |
| --- | --- | --- | --- | --- |
| *AtPDS3* | phytoene desaturase 3 | AT4G14210 | *A. thaliana* | 1701 |
| *AtWUS* | WUSCHEL | AT2G17950 | *A. thaliana* | 876 |
| *AtWOX5* | WUSCHEL related homeobox 5 | AT3G11260 | *A. thaliana* | 549 |
| *AtRHD6* | ROOT HAIR DEFECTIVE 6 | AT1G66470 | *A. thaliana* | 897 |
| *AtACTIN2* | ACTIN2 | AT3G18780 | *A. thaliana* | 1134 |
| *NbPDS* | phytoene desaturase | DQ469932 | *N. benthamiana* | 1761 |
| *NbWUS* | WUSCHEL | Niben101Scf03735g01005 | *N. benthamiana* | 936 |
| *NbWOX5* | WUSCHEL related homeobox 5 | Niben101Scf01337g03002 | *N. benthamiana* | 474 |
| *NbEF1a* | elongation factor 1-alpha | Niben101Scf04639g06007 | *N. benthamiana* | 1344 |
| *MeLRR1* | nucleotide-binding site (NBS) and leucine-rich repeat (LRR) domain, NBS-LRR1 | Manes.11G053000 | *M. esculenta* | 3063 |
| *MeLRR2* | nucleotide-binding site (NBS) and leucine-rich repeat (LRR) domain, NBS-LRR2 | Manes.03G071700 | *M. esculenta* | 2784 |
| *MeLRR3* | nucleotide-binding site (NBS) and leucine-rich repeat (LRR) domain, NBS-LRR3 | Manes.13G036800 | *M. esculenta* | 3393 |
| *MeLRR4* | nucleotide-binding site (NBS) and leucine-rich repeat (LRR) domain, NBS-LRR4 | Manes.07G107800 | *M. esculenta* | 3543 |
| *MeEF1a* | elongation factor 1-alpha | AF041463 | *M. esculenta* | 1350 |

**Supplementary Table S2** Specific primers for *in vitro*-synthesized dsRNA in this study

| **Gene**  **(GenBank Number)** | **Primer name** | **Sequence (5'-3')** | **dsRNA length**  **(bp)** |
| --- | --- | --- | --- |
| *NbPDS*  (DQ469932) | NbPDS612F-T7-F | TAATACGACTCACTATAGGGGTTCAGCCGCTTTGATTT | 195 |
|  | NbPDS806R-T7-R | TAATACGACTCACTATAGGGTGCTTTCTCATCCAGTCC |  |
| *NbWUS*  (Niben101Scf03735g01005.1) | NbWUS-114-T7-F | TAATACGACTCACTATAGGGCAGAATGGAAACTATGGTTATGG | 368 |
|  | NbWUS-481-T7-R | TAATACGACTCACTATAGGGCCAAGTTATGATCATCTGAACGG |  |
| NbWOX5  (Niben101Scf01337g03002) | NbWOX5-84-T7-F | TAATACGACTCACTATAGGGTCCCACGAATGAGCAAGTAAAAG | 294 |
|  | NbWOX5-377-T7-R | TAATACGACTCACTATAGGGAGTCTCTTTTACTCTTTCTGGTTTT |  |
| *AtACTIN2*  (AT3G18780) | AtACTIN2-219-T7-F | TAATACGACTCACTATAGGGAACTGTTGAAGTTAGATTGAATC | 232 |
|  | AtACTIN2-450-T7-R | TAATACGACTCACTATAGGGCAAAAAGAGTTCAATACAGTCAA |  |
| *AtPDS3*  (AT4G14210) | AtPDS-774-T7-F | TAATACGACTCACTATAGGGCGACGAGGTGTTTATTGCC | 283 |
|  | AtPDS-1056-T7-R | TAATACGACTCACTATAGGGGACAGTGCTTCCATTAGTGAG |  |
| *AtWUS*  (AT2G17950) | AtWUS-413-T7-F | TAATACGACTCACTATAGGGCTGCTAATTCCGTCAACGTTAAA | 314 |
|  | AtWUS-726-T7-R | TAATACGACTCACTATAGGGACATTCTTCTTCGTCTTGATGAC |  |
| *AtRHD6*  (AT1G66470) | AtRHD6-144-T7-F | TAATACGACTCACTATAGGGGAGCAGTAGCACGATGAAT | 151 |
|  | AtRHD6-294-T7-R | TAATACGACTCACTATAGGGTATGGCTGGAGGAGGAAAG |  |
| *AtWOX5*  (AT3G11260) | AtWOX5-97-T7-F | TAATACGACTCACTATAGGGAAGATATTGACTGATCTGTTTCG | 272 |
|  | AtWOX5-368-T7-R | TAATACGACTCACTATAGGGAGTTGTAATGTCTCTATCACCTT |  |
| *MeLRR1*(Manes.11G053000) | MeLRR1-614-T7-F | TAATACGACTCACTATAGGGTGCTAAGAGATGGCCCCGA | 137 |
|  | MeLRR1-750-T7-R | TAATACGACTCACTATAGGGAGCCACATCTTTGAAATCTCC |  |
| *MeLRR2*  (Manes.03G071700) | MeLRR2-201-T7-F | TAATACGACTCACTATAGGGCTGGGAGGGAAATGATTGC | 177 |
|  | MeLRR2-377-T7-R | TAATACGACTCACTATAGGGAGACGACTCAAGGAAGAACTA |  |
| *MeLRR3*  (Manes.13G036800) | MeLRR3-955-T7-F | TAATACGACTCACTATAGGGACTGTTCCTGCTCATCATCT | 143 |
|  | MeLRR3-1097-T7-R | TAATACGACTCACTATAGGGCCTTGGCACTTCCTCATTATT |  |
| *MeLRR4*  (Manes.07G107800) | MeLRR4-1265-T7-F | TAATACGACTCACTATAGGGGTTGCTTTGCTTACTGTGC | 117 |
|  | MeLRR4-1381-T7-R | TAATACGACTCACTATAGGGCCATCTCTTTATCTGTAGCGT |  |

The underlined sequences are the T7 promoter sequence (5'-TAATACGACTCACTATAGGG-3').

**Supplementary Table S3** Primers used in qRT-PCRs in this study

| **Gene**  **(GenBank number)** | **Primer name** | **Sequence**  **(5'**–**3')** | **length (bp)** |
| --- | --- | --- | --- |
| *NbPDS*  (DQ469932) | qNbPDSF | TGCTTTTGTGTTTGCCACTC | 199 |
|  | qNbPDSR | AGCGTACACACTGAGCAACG |  |
| *NbWUS* (Niben101  Scf03735g01005) | qNbWUS-F | CTCCAGTTTCTGCAACATCAAA | 113 |
|  | qNbWUS-R | GAGTAAGTTCAAGAGAAGCCCT |  |
| NbWOX5 (Niben101Scf01337g03002) | qNbWOX5-F | CTGCAACTCTTTCCACTGAA | 148 |
|  | qNbWOX5-R | GGAAGCTTAAACGCAGATCC |  |
| *NbEF1a* (Niben101  Scf04639g06007) | qNbEF1aF | AGAGGCCCTCGGACAAGC | 132 |
|  | qNbEF1aR | TGGGACCAAAAGTCACAA |  |
| *AtPDS3*  (AT4G14210) | qAtPDSF | ATCCTAACCGGTCAATGCTG | 217 |
|  | qAtPDSR | CGACATGGTTCACAGTTTGG |  |
| *AtWUS*  (AT2G17950) | qAtWUS-F | GCAAGAACGTCTTTTACTGGTT | 135 |
|  | qAtWUS-R | GATGATAATGATCGTTAGCCGC |  |
| *AtWOX5*  (AT3G11260) | qAtWOX5-F2 | TCCGGTGAATTCATTTGAAGA | 161 |
|  | qAtWOX5-R2 | GAAGATCTAATGGCGGTGGA |  |
| *AtRHD6*  (AT1G66470) | qAtRHD6-F | CATGAGCTACGGCTTCACAA | 168 |
|  | qAtRHD6-R | TTGTTTCCAGCGGATTTAGG |  |
| *AtACTIN2*  (AT3G18780) | QAtACTIN2F | AAGCTCTCCTTTGTTGCTGTT | 138 |
|  | QAtACTIN2R | GACTTCTGGGCATCTGAATCT |  |
| *MeLRR1*  (Manes.11G053000) | qMeLRR1F | GTAGAGAGAGTGATCCGTGATG | 105 |
|  | qMeLRR1R | CAGCCGATCAACTTCATTTAGG |  |
| *MeLRR2*  (Manes.03G071700) | qMeLRR2F | TGCAGCTGTCAATGGAATTAAC | 110 |
|  | qMeLRR2R | GCTGATTTCTTGACAGGTTCAG |  |
| *MeLRR3*  (Manes.13G036800) | qMeLRR3F | CAAGTTAAAAACGCTTCGCATC | 121 |
|  | qMeLRR3R | CAAATTAGGACACTCCCTGACT |  |
| *MeLRR4*  (Manes.07G107800) | qMeLRR4F | GCTTTGTATGAAGCCGATGATT | 96 |
|  | qMeLRR4R | CATCTGATCTCCAAAGGTACGA |  |
| *MeEF1a*  (AF041463) | QMeEF1aF | TGAACCACCCTGGTCAGATTGGAA | 139 |
|  | QMeEF1aR | AACTTGGGCTCCTTCTCAAGCTCT |  |
